# Engineering Cobalt‐Based Thin Films for Fischer–Tropsch Synthesis

**DOI:** 10.1002/smll.75333

**Published:** 2026-08-24

**Authors:** Muhammad Hamid Raza, Avela Kunene, Alexander Steigert, Athanasios Skaltsogiannis, Catalina E. Jiménez, Florian Ruske, Ali Shan Malik, René Schwiddessen, Judith Helena Mettke, Johannes Frisch, Regan G. Wilks, Erik Reichelt, Marcus Bär, Rutger Schlatmann, Daniel Amkreutz

**Affiliations:** ^1^ PVcomB Helmholtz‐Zentrum Berlin für Materialien und Energie GmbH (HZB) Berlin Germany; ^2^ Department of Interface Design HZB Berlin Germany; ^3^ Energy Materials In‐situ Laboratory Berlin (EMIL) HZB Berlin Germany; ^4^ Department Active Materials and Interfaces for Stable Perovskite Solar Cells Helmholtz‐Zentrum Berlin für Materialien und Energie (HZB) Berlin Germany; ^5^ Department Structure and Dynamics of Energy Materials Helmholtz‐Zentrum Berlin für Materialien und Energie (HZB) Berlin Germany; ^6^ Fraunhofer Institute for Ceramic Technologies and Systems IKTS Dresden Germany; ^7^ Helmholtz‐Institute Erlangen‐Nürnberg for Renewable Energy (HI ERN) Department of X‐ray Spectroscopy at Interfaces of Thin Films Berlin Germany; ^8^ Friedrich‐Alexander‐Universität Erlangen‐Nürnberg (FAU) Department of Chemistry and Pharmacy Erlangen Germany

**Keywords:** atomic layer deposition, cobalt, fischer‐tropsch synthesis (FTS), magnetron sputtering, physical vapour deposition (PVD), plasma enhanced chemical vapour deposition, thin films

## Abstract

We report on the synthesis of cobalt‐based thin films via magnetron sputtering at varying conditions, including sputtering pressure, reactive gas composition, substrate temperature, and post‐deposition annealing. The produced thin films exhibit diverse morphologies, crystalline phases, and oxidation states, such as metallic cobalt (Co^0^) and cobalt oxides (CoO and spinel‐Co_3_O_4_). Two thin‐film catalyst architectures, based on Co^0^ (TFCat1) and Co_x_O_y_ (TFCat2), on Si/Al_2_O_3_/SiO_2_ support stacks are further developed as analogues of commercial powder‐based Fischer–Tropsch synthesis (FTS) catalysts. The phase evolution in both of these model catalyst systems is investigated in situ under FTS‐relevant reduction conditions using synchrotron‐based grazing‐incidence x‐ray diffraction. Both catalysts (TFCat1 and TFCat2) were tested for their FTS catalytic performance at comparable operating conditions. A lined‐out CO conversion level of *ca*. 4.6% and 4.9% was obtained for TFCat1 and TFCat2, respectively. These catalysts exhibited distinct steady‐state product distributions: TFCat1 showed enhanced selectivity toward kerosene‐range hydrocarbons, while TFCat2 produced a wider hydrocarbon distribution extending to C_27_. Moreover, Cobalt thin‐film model catalyst (TFCat1) shows significantly reduced dewetting compared to its oxide counterpart (TFCat2) under FTS operating conditions. The well‐defined and easy to tune cobalt‐based thin films provide a robust foundation for the rational scale‐up of next‐generation catalysts.

## Graphical abstract

Cobalt thin films with tailored crystalline phases and oxidation states serve as well‐defined model Fischer–Tropsch catalysts. In situ synchrotron GIXRD reveals distinct reduction behaviour and phase evolution, while catalytic testing demonstrates phase‐dependent hydrocarbon selectivity and improved structural stability. These model thin‐film catalysts provide a robust platform for the rational design and scale‐up of next‐generation Fischer–Tropsch synthesis (FTS) catalysts.

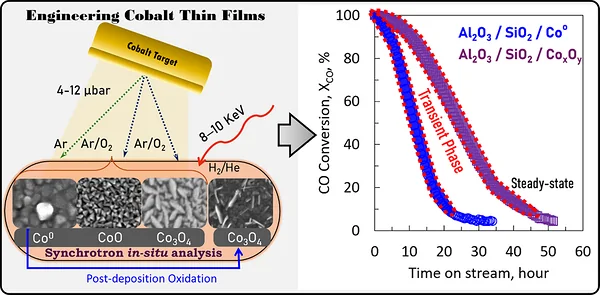

## 1 Introduction

Thin films are one of the cornerstones of contemporary materials science, providing a platform for nanoscale‐structured materials with precise engineering capabilities. Thin films have become a necessity in a broad range of technological fields due to their ability to tailor physical and chemical properties [1, 2]. Among various applications, their implementation in catalytic systems, and most notably in heterogeneous catalysis, has raised considerable interest [3, 4, 5, 6, 7]. The natural attributes of thin‐film systems, such as the ability to tune the composition and well‐defined surface structures on different support materials and substrates allow for catalytic structures that are not readily available in bulk or nanoparticle‐based materials [7, 8]. Such systems allow for maximum utilization of catalytically active moieties, expedited activation under reaction environments, and in many cases, increased selectivity toward desired end‐products [9, 10, 11, 12, 13, 14]. Thin films in heterogeneous catalysis offer special opportunities for rational catalyst development by controlling the chemical environment of the active site [9, 10, 11, 12, 15]. Such well‐defined surfaces make these systems ideal model platforms for testing catalytic materials.

A key parameter in designing efficient thin‐film catalysts is the control of morphology and, critically, the oxidation state of the catalytically active material [10, 16, 17]. The catalytic behaviour of thin films is highly sensitive to these properties, as they influence surface energy, adsorption properties, reducibility, and active site availability [4, 10, 13]. Despite some progress in applying thin films for thermal catalysis, a comprehensive understanding of how synthesis parameters govern morphology, crystalline phase, and chemical structure remains underdeveloped [9, 18].

Iron‐ and cobalt‐based catalysts are widely used in commercial‐scale Fischer–Tropsch synthesis (FTS) [19]. Cobalt remains the most studied FT catalyst due to its highest activity and selectivity toward the formation of long‐chain hydrocarbons, and improved thermal stability compared to iron‐based catalysts [19]. Cobalt catalysts are employed at low temperatures, consume more H_2_, and predominantly form straight‐chain paraffin‐key constituents of jet fuel‐without the production of additional CO_2_ [19, 20]. The catalytic activity is significantly governed by the nature of the cobalt chemical structure and crystalline phases; metallic cobalt is usually most active in FTS. CoO and Co_3_O_4_ can behave as precursors or vary in reducibility and dispersion under activating conditions [19, 21]. Cobalt's polymorphs (*hcp*‐ or *fcc*‐Co^0^) can influence the overall intrinsic activity in FTS with *hcp‐*Co^0^ generally exhibiting higher activity than *fcc*‐Co^0^ [22, 23, 24, 25]. Thus, also the optimization of the chemical structure and crystallographic phase of thin‐film catalysts is critical for the overall FTS catalyst performance [19, 25, 26, 27, 28, 29].

Physical vapor deposition (PVD) is an umbrella term that generally includes cost efficient and scaleable thin‐film preparation methods, such as thermal evaporation, electron‐beam evaporation, and sputtering [30]. PVD methods are sought for reasons of cost‐effectiveness, scalability, reproducibility, and material compatibility with wide‐ranging systems. Magnetron sputtering (MS) is known for being able to deposit films with specially tailored phase composition and morphology by modulating sputtering pressure, sputtering power, target and substrate geometry, gas mixture, substrate biasing, and temperature [22, 30, 31, 32]. Use of reactive and non‐reactive MS for the preparation of catalysts represents a highly selective means of preparing model catalytic systems with preprogrammed active phases, shaped nanostructures, and low contamination levels; hence is often the technique of choice for systematic phase–structure–property relation studies [22, 30, 31, 33, 34, 35]. While the concept of thin‐film catalysts is emerging, the underlying fabrication techniques are already fully scalable, widely adopted, and compatible with industrial‐scale production [8, 22, 30, 31, 32].

In this paper, we deposited cobalt‐based thin films with well‐controlled morphologies, crystalline phases and chemical structure, such as Co^0^, CoO, and *spinel‐*Co_3_O_4_, by tuning the direct current (DC) reactive and non‐reactive MS deposition conditions. Structural and morphological characterizations are performed by grazing incidence x‐ray diffraction (GIXRD), scanning and transmission electron microscopy (SEM/TEM), and x‐ray photoelectron spectroscopy (XPS). Moreover, thin‐film catalyst systems with either Co or Co_x_O_y_ on Si wafer/Al_2_O_3_/SiO_2_ and Aluminium foil/Al_2_O_3_/SiO_2_ support stacks, mimicking the structure of commercial standard FTS powder‐based catalysts, but with ultra‐low cobalt loading, are synthesized and tested under FTS. The dynamics of the phase transformation and its relationship to the stability of the catalyst and retention of the phases during FTS reduction step were explored using synchrotron‐based in situ GIXRD studies in a reducing environment. The characterization and catalytic performance provide an insight into the evolution of the chemical structure and crystalline phases of cobalt thin films and their subsequent influence on the catalytic activity and product distribution, and suggest guidelines for future next‐generation catalyst designs.

## 2 Material and Methods

### 2.1 Thin‐Film Depositions

Cobalt and cobalt oxide thin films were deposited using a ultra‐high vacuum (UHV) system manufactured by PREVAC with sputter sources provided by Thin Film Consulting. A 99.99% purity metallic cobalt target (2 inch diameter, purity 4N), was employed as material source. The discharge power was fixed at 100 W throughout the depositions. The base pressure in the vacuum chamber was maintained at 1 × 10^−8 ^mbar, while the sputtering pressure was varied between 4–12 × 10^−3 ^mbar. The deposition pressure was controlled by varying the process gas flow rates of high‐purity argon (6N) and oxygen (6N). For reactive MS, the Ar:O_2_ ratio was varied as specified in, Table S1. Films were deposited on the single‐side polished Si(100) wafers. The substrate temperature was maintained at room temperature or, where specifically indicated, heated to 500 °C. The samples deposited by non‐reactive and reactive MS are referred to as NR and R samples, respectively.

Al_2_O_3_ films were deposited by thermal atomic layer deposition (ALD) in a commercial TFS‐200 reactor (BENEQ). Trimethylaluminium (TMA) and water (DI) were used as precursors, and deposition was performed at 200°C.

SiO_2_ films were deposited by plasma‐enhanced chemical vapor deposition (PECVD) at 300°C using silane (SiH_4_) and nitrous oxide (N_2_O) precursors. The process was carried out in a PlasmaPro 100 PECVD “nanofab” in cluster configuration from Oxford Instruments Plasma Technology.

Both, Al_2_O_3_ and SiO_2_ oxide layers, were only deposited for the model thin‐film catalysts (TFCat1 and TFCat2) onto the Si wafers (for structural characterizations) and on aluminium foil (for FTS testing). These oxide layers function as dense thin‐film supports and buffer layers, and do not provide porosity for Co penetration to the Co/Co_x_O_y_ films deposited by physical vapour deposition, *cf*. discussion below with Figures 5 and 6 [6, 36, 37].

### 2.2 Characterizations

Film thicknesses were estimated by x‐ray reflectivity (XRR) and further confirmed by using a J.A. Woollam spectroscopic ellipsometry (SE) for the oxide films. The metallic films exceeded the thickness range suitable for reliable SE; therefore, only the XRR values are reported for these samples. The measured film thicknesses of the TFCat1 and TFCat2 samples were further corroborated directly by cross‐sectional scanning transmission electron microscopy (STEM) analysis. Crystal structures were characterized using grazing incidence x‐ray diffraction (GIXRD) at an incidence angle ω of 0.5° on a PANalytical diffractometer equipped with a Cu K_α_ source (λ = 1.5406 Å). Surface morphologies were examined using a Zeiss Merlin Gemini 2 scanning electron microscope with an accelerating voltage of 5 kV. High‐angle annular dark‐field scanning transmission electron microscopy (HAADF‐STEM), bright‐field scanning transmission electron microscopy (BF‐STEM) and HRTEM were carried out on a FEI Talos F200S S/TEM operated at 200 kV. Energy‐dispersive x‐ray spectroscopy (EDX) spectra and elemental maps were obtained using the integrated SuperX EDS detector. Elemental maps of Co, O, Si, and Al were acquired using the respective K_α_ fluorescence lines to resolve their spatial distribution. TEM measurements were performed on cross sections of Focused Ion Beam (FIB) prepared sample lamellas. The analysis of the electron microscopy data was performed using Velox 2.6 software.

X‐ray photoelectron spectroscopy (XPS) measurements were carried out under ultrahigh vacuum conditions (10^−10^–10^−9^ mbar pressure) using a monochromatized Al K_α_ source from SPECS (XR 50 M x‐ray source with a FOCUS 500 x‐ray monochromator) providing an excitation energy of 1486.58 eV. The samples were transferred directly from the sputtering chamber to the XPS through an ultra‐high‐vacuum (UHV) transfer line without exposure to air. The kinetic energy of photoemitted electrons was determined using a hemispherical electron analyzer from SPECS (PHOIBOS 150) equipped with a channeltron detector (MCD‐9). For each sample a survey and detail spectra of the most prominent core levels were acquired, with a pass energy of 100 and 20 eV, respectively. The instrument was regularly calibrated by referencing the Fermi edge position of a clean Au foil to a binding energy of 0.0 eV. As the studied samples were adequately conductive (and no sample charging was observed) the data was analysed without energy scale correction.

The phase transformation of as‐deposited Co and Co_x_O_y_ films under reduction conditions (3% H_2_ in He) was in situ studied in a temperature range between 330°C and 600°C using synchrotron‐based GIXRD performed at the KMC‐2 beamline of BESSY II operated by HZB. The KMC‐2 beamline provides highest photon flux in the range of 8–10 keV. For these experiments, a photon energy of 8048 eV (λ = 1.5406 Å) combined with a 2D Bruker Vantec 2000 detector, enabled a time resolution of 79 min per diffractogram scanning the 2θ range 28°–82°. The incident beam had a cross‐section of 1 × 0.15 mm^2^ (width x height) which at an incidence angle ω of 1°, illuminated ca. 10 × 1 mm^2^. An x‐ray–transparent Anton Paar DHS 1100 furnace was used to heat the samples under reducing or in oxidizing environments. This setup allows real‐time monitoring of phase evolution in cobalt‐based thin films at elevated temperatures. The experimental design of the heating treatments during the in situ GIXRD is visualized in Figure S1.

### 2.3 Fischer‐Tropsch Synthesis Catalysis

To measure the FTS activity two model thin film catalyst samples were prepared onto a 10 × 10 cm^2^ aluminum foil (Thermo scientific, Puratronic 0.1 mm (0.004 in) thick, Purity: 99.997% (metal basis)) substrate, resulting in Al foil/Al_2_O_3_/SiO_2_/Co^0^ (TFCat1) and Al foil/Al_2_O_3_/SiO_2_/Co_x_O_y_ (TFCat2) stacks. A total of ca. 1800 mg of TFCat1 and TFCat2 catalysts was cut into small pieces and mixed with 0.78 g of SiC (300 mesh), yielding a Catalyst/SiC mass dilution factor of 2. The catalyst and SiC mixture were then loaded into a fixed‐bed reactor, described in Ref. [38]. The loaded catalyst was reduced in situ with flowing H_2_ at 200 mL(NTP)/min for 16 h at 350°C. The residual H_2_ from the reduction protocol was evacuated with N_2_ at 200 mL(NTP)/min, while decreasing the temperature from 350°C to 250°C. At 250°C, the reactor was pressurised to 20 bar using N_2_. At this point (t = 0) the N_2_ is switched with the reaction mixture (a total gas flow rate of 50 mL/min using a syngas premix (H_2_/CO in N_2_) with an H_2_:CO ratio of 1.7). The FTS catalytic performance of the synthesized thin‐film catalysts was then tested (at 250°C, 20 bar) in a transient state until it reached steady‐state. The gas phase of the product was analysed via microGC (Inficon Fusion). The liquid product was analysed by adding 1 mL of Toluene and 1 mL of water to the sample. Both phases were analysed in a GC (Agilent 7980B). Further details are given in Ref. [38]. In the aqueous phase, no products could be detected.

## 3 Results and Discussion

DC reactive and non‐reactive MS in argon and argon‐oxygen environments were used to deposit cobalt and cobalt oxide thin films with controlled morphology and structure (see Table S1 for details). Figures 1a,b,c and 2a show the SEM micrographs and GIXRD patterns, respectively, of the films deposited by non‐reactive MS in argon. All the samples exhibit continuous and homogeneously covering films. Varying the sputtering pressure between 4–12 × 10^−3^ mbar did not significantly affect the morphology of the deposited films. However, the sample deposited at 4 × 10^−3^ mbar (NR1) exhibited single‐phase metallic cobalt with a hexagonal close‐packed (*hcp*) structure, with apparently larger crystallite sizes compared to the samples deposited at higher sputtering pressures (NR2, NR3), as the FWHM of the *hcp*‐ Co^0^ *002* reflection increased from sample NR1 to NR3, with 0.35, 0.67, and 0.71, respectively (Figure 2a). A minor contribution of *fcc*‐Co^0^ and CoO is observed in samples deposited at higher sputtering pressures (NR2 and NR3). Since these samples were measured after prolonged exposure to air, the observed CoO is due to oxidation that occurred during air exposure rather than during film deposition. In contrast, measurements performed immediately after sample preparation show no involvement of oxidized phases (data not shown here). Furthermore, no significant oxygen signal is detected in the XPS spectra of the as‐deposited state (*cf*. XPS discussion below), confirming the absence of oxidation during deposition. As sample NR1 deposited at 4 × 10^−3^ mbar exhibited single‐phase *hcp*‐Co^0^ and a higher crystallinity, this sputter pressure was selected for subsequent experiments. Post‐deposition annealing in UHV at 600°C for 1 h of sample NR1 improved the crystallinity of the deposited films while maintaining the same phase and orientation (Figure S2). Post‐deposition annealing in an O_2_ environment for 1 h at 500°C of the film deposited at a sputter pressure of 4 × 10^−3^ mbar produced a less uniform film with a more diffusive particle distribution, indicating a partial oxidation of the film surface (Figure 1b, NR4). The thickness of the film increased from 39 to 69 nm, likely due to the incorporation of oxygen into the crystal lattice. The GIXRD pattern indicates that the film is composed of a mixture of CoO (*fcc*, dominant) and Co_3_O_4_ (*spinel‐*, minor) phases (Figure 2a, NR4). Raising the substrate temperature to 500°C during deposition under the same conditions as above resulted in *hcp*‐Co^0^ deposition with an increase in particle size with uniform distribution, and a film thickness of 50 nm (NR5, Figures 1c and 2a).

**FIGURE 1:**
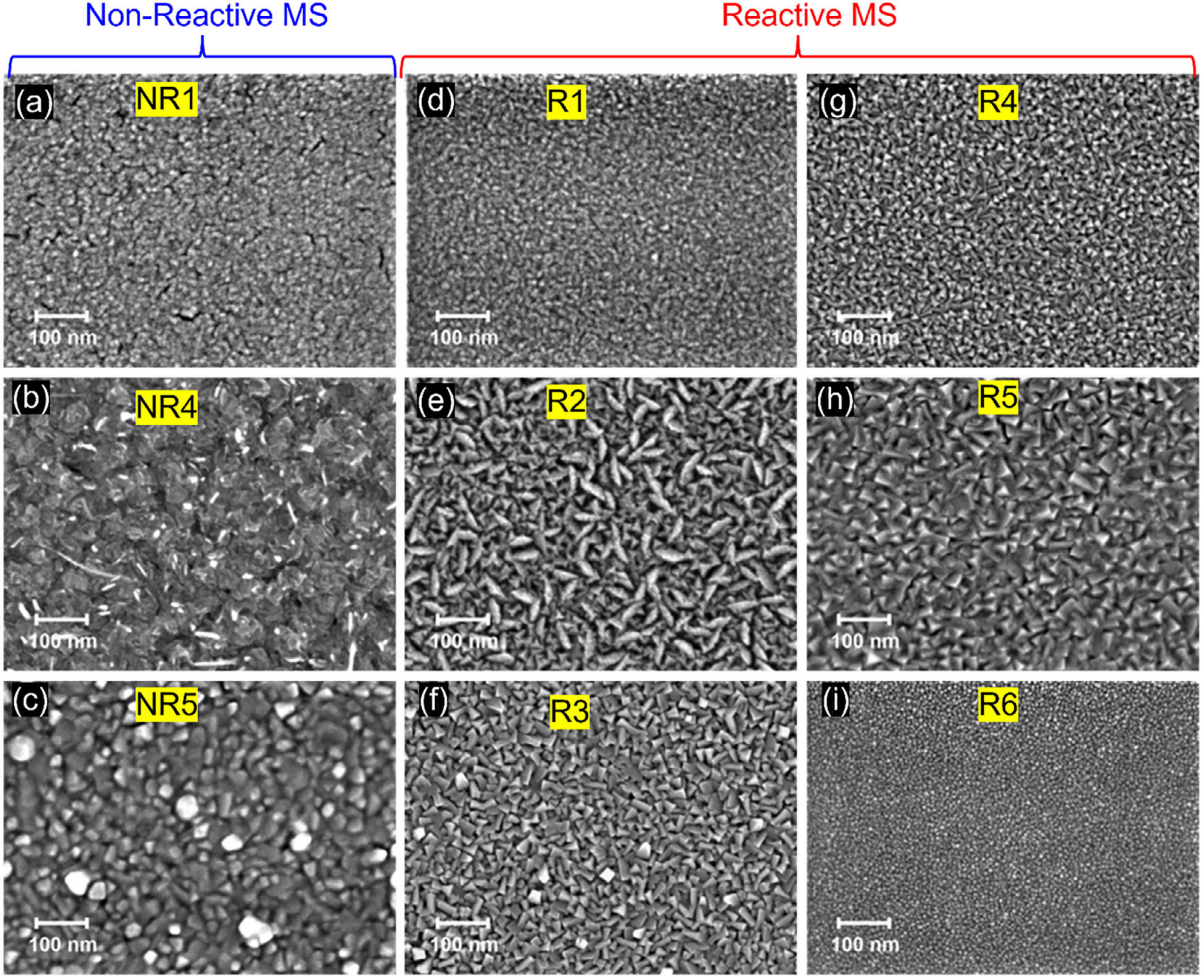
SEM micrographs of cobalt‐based thin films deposited under different conditions as mentioned in Table S1: (a) NR1; (b) NR4; (c) NR5; (d) R1; (e) R2; (f) R3; (g) R4; (h) R5; (i) R6. All images are shown at same magnification.

**FIGURE 2:**
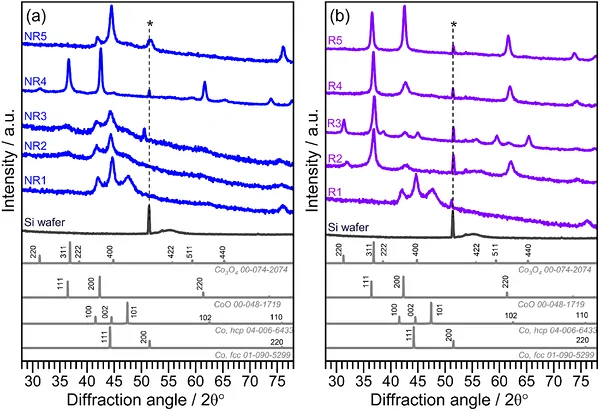
GIXRD diffractograms of cobalt‐based thin films deposited by (a) non‐reactive and (b) reactive MS.

DC reactive magnetron sputtering of cobalt in a 10:1 mixture of Ar:O_2_ at RT gave significantly different results based on the sputtering pressure (contrary to the non‐reactive cobalt sputtering). At 4 × 10^−3^ mbar, *hcp*‐Co^0^ with a film thickness of 46 nm (similar to what we deposited using non‐reactive sputtering, *i.e*., 40 nm) was obtained (Figures 1d and 2b, R1). With an increase in the sputtering pressure to 12 × 10^−3^ mbar, a film composed of cobalt oxide with a typical needle/rod‐type morphology with a film thickness of 90 nm was obtained (Figure 1e, R2) [35]. The GIXRD pattern shows mainly *fcc*‐CoO crystallographic structure with a minor contribution of *spinel*‐Co_3_O_4_ (Figure 2b, R2). When the substrate temperature was increased to 500°C while maintaining the pressure at 12 × 10^−3^ mbar (Ar:O_2_ = 10:1), the film exhibited a pyramid‐like particulate morphology with a thickness of 74 nm and consisted of a mixture of spinel Co_3_O_4_ and *fcc*‐CoO phase (Figures 1f and 2b, R3). This characteristic faceting is anticipated to have a significant effect on surface reactivity especially in catalysis applications [13, 26].

In the following set of depositions, the O_2_ concentration in the Ar:O_2_ mixture was increased (10:1―1:1 of Ar:O_2_). The film deposited using Ar:O_2_ (2:1) at 4 × 10^−3^ mbar, both at RT and 500°C, consist of CoO (R4 and R5, Figure 2b, *cf*. XPS below). Both films exhibit pyramid‐like morphologies with larger particle sizes at 500°C than at RT. The thicknesses of the films for samples R4 and R5 were derived to be 85 and 97 nm, respectively. Further increasing the oxygen to Ar:O_2_ (1:1) substantially decreased the deposition rate and produced a fine particulate film with a significantly reduced thickness of 12 nm (Figure 1i). No diffraction peaks could be observed for this sample, either due to a lower film thickness (not sufficient for GIXRD even at 1°) or indicating an XRD‐amorphous cobalt oxide. Overall, both non‐reactive and reactive MS under defined conditions showed homogeneous, well‐covering films deposited onto the Si wafers, as shown in the cross‐sectional and top‐view SEM micrographs of representative samples (Figure 3).

**FIGURE 3:**
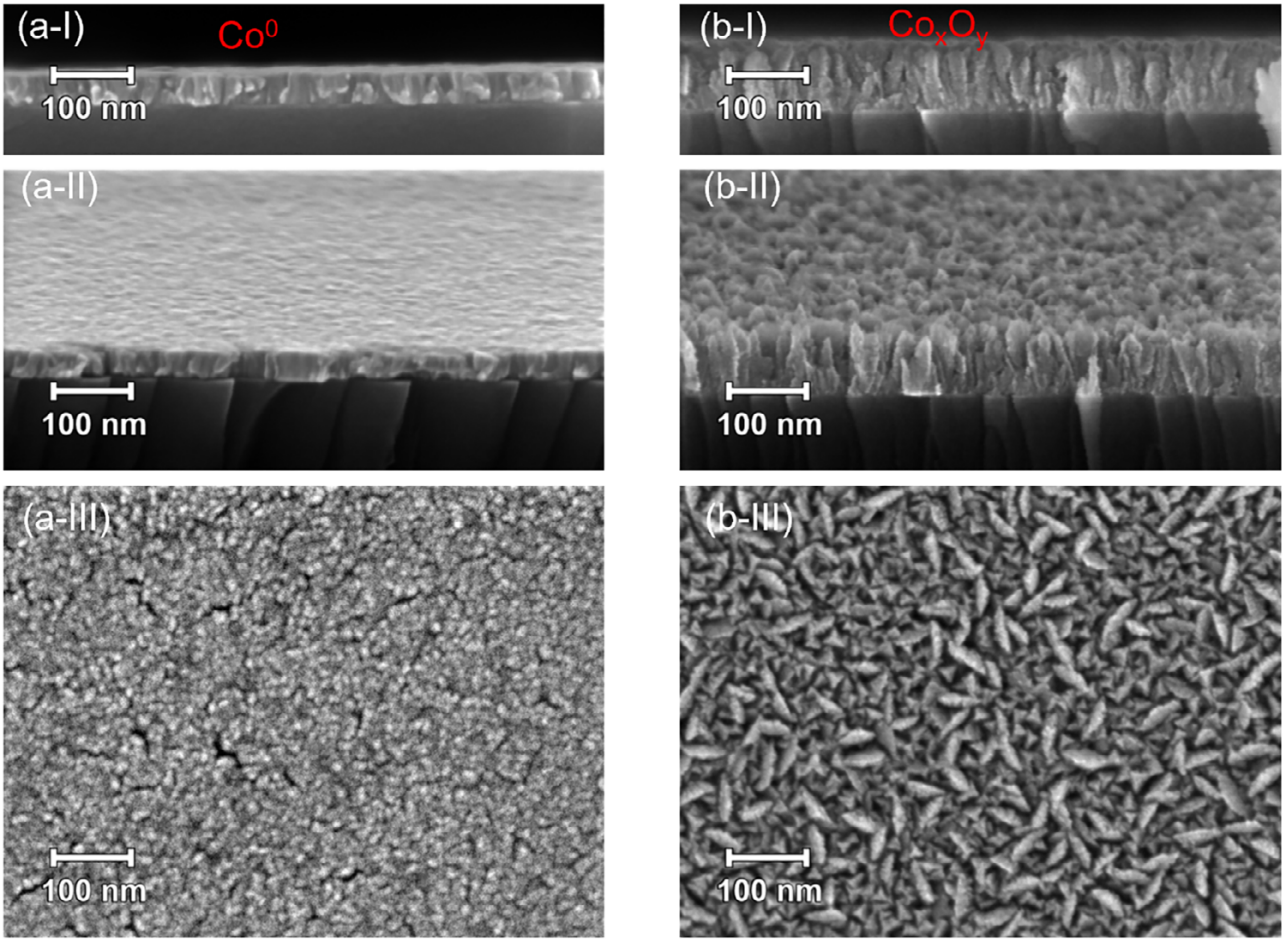
Cross‐sectional and top‐view SEM micrographs of cobalt‐based thin films deposited by (a) non‐reactive MS at RT, 4 × 10^−3^ mbar in Ar (NR1) and (b) reactive MS at RT in Ar:O_2_ = 10:1, 12 × 10^−3^ mbar (R2).

XPS provided information on the surface chemical composition of the samples, with the core levels of interest having photoelectron inelastic mean free path in Co of approximately 1–1.5 nm [39, 40]. Figure 4 shows the Co 2p spectra of the three analysed samples in comparison with reference spectra digitized from Biesinger et al. [41]. After deposition, the samples were transferred directly from the sputtering chamber to the XPS through an ultra‐high‐vacuum (UHV) transfer line without exposure to air. Sample NR1 was found to be composed mostly of metallic Co (with minor O and N contaminations), with an O/Co atomic ratio equal to approximately 0.04 (see details on quantification in SI in conjunction with the discussion of Figure S3). No photoemission signal from the substrate is visible in the survey spectrum (Figure S4), corroborating the SEM images in Figures 1a and 3a. The main Co 2p peak position at 778.1 eV and its characteristic asymmetric peak shape indicate the metallic character of the sample (Figure 4), in agreement with measurements reported in the literature [41] and corroborating the GIXRD in Figure 2a. Reasonable fitting of the Co 2p peak was possible assuming that the sample is purely metallic (see SI). For sample R5, only Co and O signals were observed in the survey spectrum (see Figure S4), and the O/Co ratio was determined to be approximately 1 (see details on quantification in SI in conjunction with the discussion of Figure S3). The Co 2p main peak is shifted by 1.9 eV compared to the metallic sample and shake‐up satellite peaks characteristic of a Co^2+^ type chemical environment are present. The O 1s core level spectrum shows a main peak at 529.5 eV attributed to Co‐O lattice oxygen species [42], while two small peaks were detected at higher binding energies, at 530.5 and 531.5 eV assigned as low‐coordinated/defective O species on CoO surface or to ‐OH species (see Figure S5). Accordingly, it was concluded that the sample is composed mainly of CoO (minor Co(OH)_2_ presence cannot be excluded), thus the Co 2p spectrum was fitted by assuming that the sample is composed only of CoO (*cf*. also in agreement with the GIXRD results in Figure 2b above). The surface composition of sample R3 was quite similar to that of R5, with an O/Co ratio equal to 0.96 (see details on quantification in SI in conjunction with the discussion of Figure S3). The Co 2p spectrum was fitted using the same fitting parameters as for the sample R5.

**FIGURE 4:**
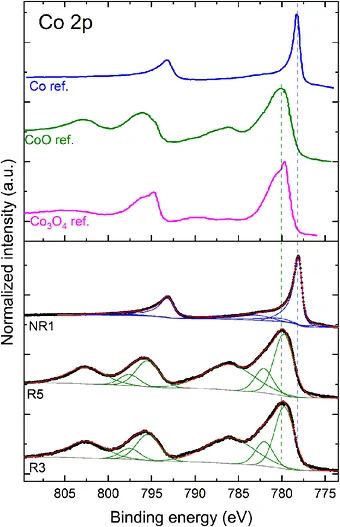
Al K_α_ excited Cobalt 2p detail spectra of the three samples (NR1, R3, and R5; bottom three spectra) and of reference compounds from literature (top three spectra) [41]. The experimentally derived spectra of this study are shown together with their fits according to Reference [41] with the blue contribution corresponding to metallic Co and the green contribution corresponding to CoO (details for the peak fitting are presented in, Tables S2 and S3). The spectra are shown vertically offset for better visibility.

The above findings show that pressure and sputtering atmosphere help to tune and define Co_x_O_y_ phases and the surface morphology. Sputtering at low pressure in pure Ar (and at Ar:O_2_, 10:1) produce *hcp* Co^0^, but raising the oxygen partial pressure tends to favour oxide phases, with pressure and temperature affecting the phase (CoO vs. Co_3_O_4_) and particle faceting. Notably, CoO (*fcc*) and Co_3_O_4_ (*spinel*) depict different morphologies, with CoO dominating as pyramid‐like particulate films, whereas Co_3_O_4_ shows needle/rod‐like morphologies [35].

The as‐synthesized DC‐sputtered, metallic Co films can be easily oxidized. Complete oxidation of approximately 40 nm thick films was achieved by annealing in air for 4 h at 350°C, with a complete structural transformation from the *hcp*‐Co^0^ to the spinel Co_3_O_4_ (Figure S6). A clear morphological transformation occurred with the oxidation such that the initially particulate film‐like surface of Co° converted into rod/needle‐like structures. Notably, the resultant morphology of the post‐deposition oxidized film differs from that of films directly deposited as cobalt oxide by reactive MS (*cf*. Figure 1d–i and Figure S6), revealing thereby the effect of the path of oxidation on the resultant microstructures. Thus, two distinct methods of synthesis can be distinguished: (i) direct Co_x_O_y_ deposition by reactive MS in Ar:O_2_ and (ii) deposition of Co^0^ with subsequent oxidization in air at 350°C. A more reproducible and phase‐pure spinel Co_3_O_4_ is obtained from the latter method, showing that the oxidation of pre‐deposited metallic cobalt offers very reproducible and better phase selectivity than direct oxide deposition by reactive MS using Ar/O_2_ plasma for the given set of parameter. The oxidation process of Co^0^ was further studied by in situ GIXRD during heating in air in an x‐ray transparent furnace. The results reveal that oxidation initiates at approximately 330°C, with the *hcp* Co^0^ reflections progressively diminished, and spinel Co_3_O_4_ peaks emerged. After ∼3.5 h at 350°C, the transformation was complete, giving a phase‐pure spinel Co_3_O_4_ structure (Figure S7). This controlled in situ study further confirmed the temperature threshold and time scale required for the full *hcp‐*cobalt‐to‐*spinel‐*Co_3_O_4_ conversion under oxidative conditions. Thermal catalysis is a key application of cobalt thin films, particularly in FTS. Thus, the phase and morphology of cobalt thin films, which are critically dependent on deposition conditions, may have direct implications for the FTS reaction [13, 26, 27]. The ability to tune particle faceting, such as pyramid‐ or needle‐like structures observed in oxide phases, offers a potential method for controlling the density of the active sites and the extent of metal‐support interaction upon activation. The transition from metallic to oxide cobalt with increasing oxygen concentration provides a means to engineer the chemical structure and crystallographic phases of the deposited films, affecting their reducibility and dispersion [23, 43].

To probe the relevance of FT catalysis, two prototype thin‐film catalyst systems were designed based on metallic cobalt and cobalt oxides. They were intended to model the commercial powder‐based FTS catalyst structures but with better control over the chemical composition and crystalline phase. Both thin‐film catalyst structures were grown on Si(*100*) wafers with an Al_2_O_3_ support layer and a SiO_2_ buffer layer (analogous to support material used in the current state of art FTS catalyst, which is being used in FTS commercial applications such as slurry phase Gas‐to‐Liquid (GTL) plants, etc) forming the following stacks: Si/Al_2_O_3_/SiO_2_/Co^0^ (TFCat1) and Si/Al_2_O_3_/SiO_2_/Co_x_O_y_ (TFCat2) [44]. About 40 nm thickness of cobalt or cobalt oxide film was deposited on the buffered substrates. The HRTEM micrographs of both the samples indicate that the films are homogeneous, conformal, and of defined thickness, in accordance with the precise deposition control of ALD (for Al_2_O_3_) [4, 6, 36], PECVD (for SiO_2_), and non‐reactive and reactive MS (for Co or Co_3_O_4_), see Figures 5a and 6a. HAADF‐STEM micrographs (Figures 5b and 6b) further confirm the conformal stacked structure, and EDX elemental mapping verifies the homogeneous distribution of Al, Si, O, and Co in the respective multilayer stacks both in TFCat1 and TFCAT2 (Figures 5c and 6c). Due to cross‐sectional TEM/EDX mapping performed on FIB‐cut thin lamellae after exposing to air, the cobalt region in both samples shows a detectable oxygen signal.

**FIGURE 5:**
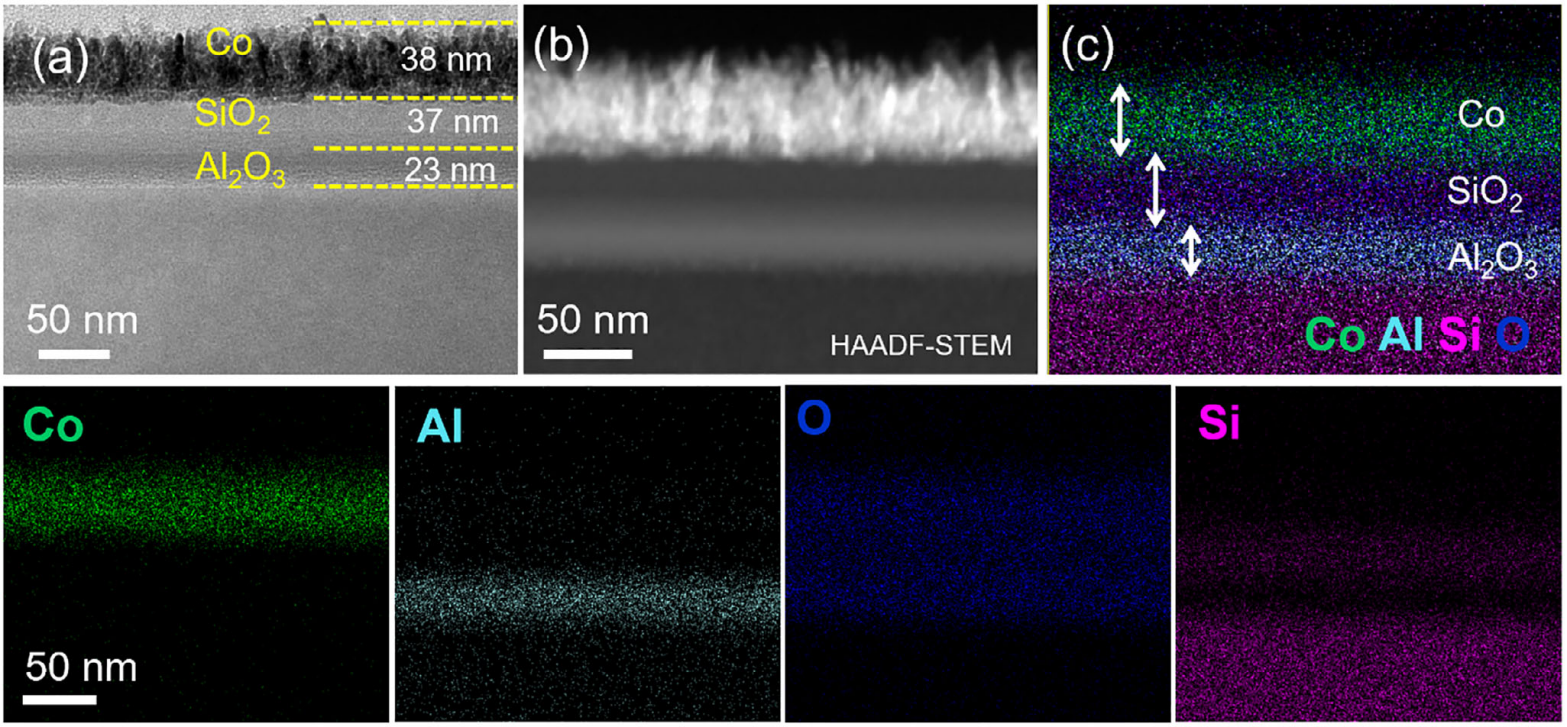
(a) Bright‐field scanning electron microscopy (BF‐TEM) micrograph, (b) high‐angle annular dark‐field scanning transmission electron microscopy (HAADF‐STEM) micrograph, and (c) energy‐dispersive x‐ray spectroscopy (EDX) combined elemental mapping of cross‐section of the Si/Al_2_O_3_/SiO_2_/Co^0^ (TFCat1) sample. Bottom panels show the corresponding individual elemental distributions for (c). The corresponding EDX spectra is shown in Figure S8a.

**FIGURE 6:**
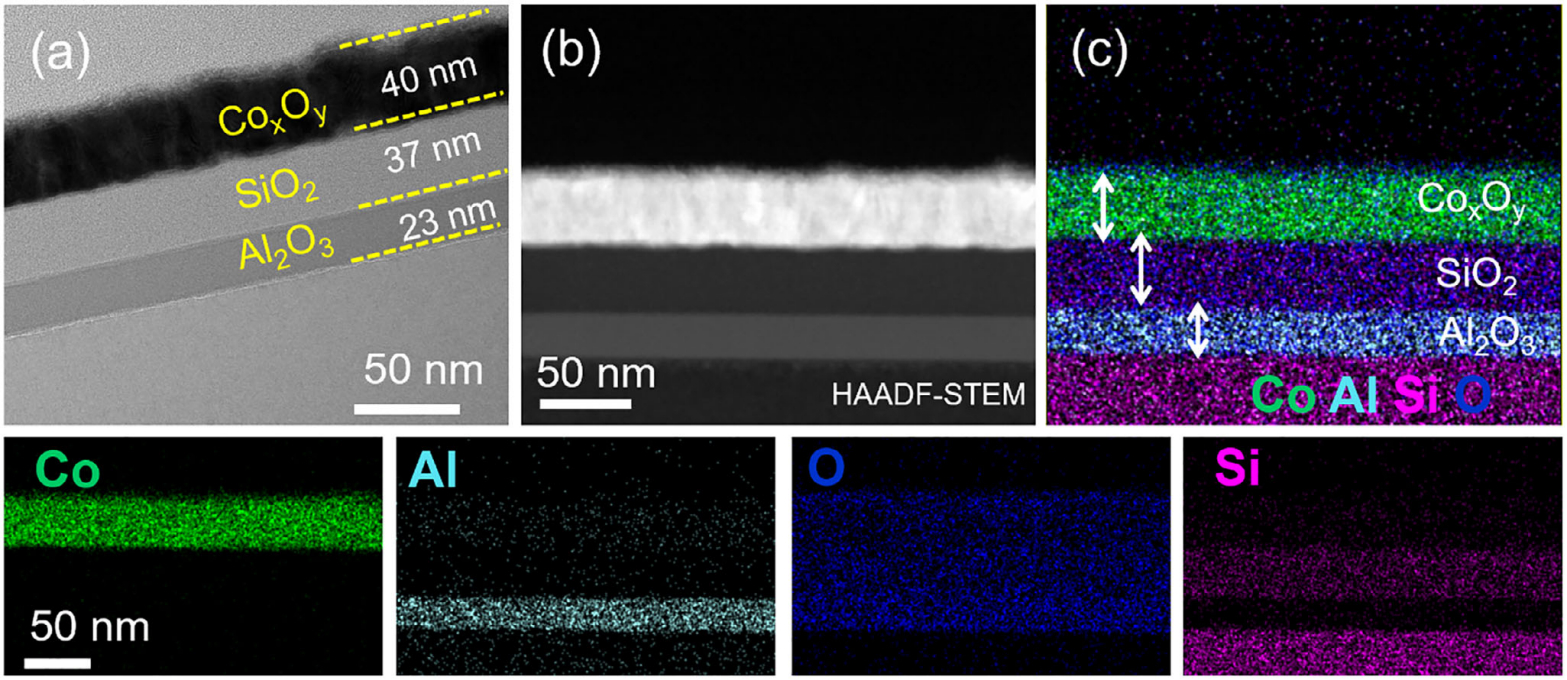
(a) Bright‐field scanning electron microscopy (BF‐TEM) micrograph, (b) high‐angle annular dark‐field scanning transmission electron microscopy (HAADF‐STEM) micrograph, and (c) energy‐dispersive x‐ray spectroscopy (EDX) combined elemental mapping of cross‐section of the Si/Al_2_O_3_/SiO_2_/Co_x_O_y_ (TFCat2) sample. Bottom panels show the corresponding individual elemental distributions for (c). The corresponding EDX spectra is shown in Figure S8b.

To assess the structural stability and phase evolution under catalytic activation conditions, in situ high‐temperature synchrotron grazing‐incidence x‐ray diffraction (HT‐GIXRD) was performed under reducing (H_2_/He) environment. For each sample, diffraction was recorded at several key stages: (i) before heating (as deposited) at room temperature (RT, in air), (ii) during heating to target temperatures under dome in transparent furnace (in situ in H_2_/He), and (iii) after cooling down to RT.

### 3.1 Phase Evolution of TFCat1

The as‐deposited TFCat1 shows *hcp*‐Co^0^ diffractions patterns only (*cf. description above*, Figure 2). No other diffraction pattern can be observed for Al_2_O_3_ or SiO_2_, in agreement with the amorphous nature of the PECVD‐SiO_2_ and ALD‐Al_2_O_3_ support films (Figure 7a) [6, 37, 45]. When heated in a reducing environment to 350°C, the intensity of the *002 hcp*‐Co^0^ reflection increased showing enhanced crystallinity. At 375°C, the preferred *hcp*‐Co structure continues to dominate, with a negligible onset formation of *fcc*‐Co^0^ reflection at approximately 51°, distinguishable diffraction transformation of *fcc*‐Co^0^ to *hcp*‐Co^0^. The principal *fcc*‐ Co^0^ *111* reflection in the range of 43°–44° overlaps with the dominant *hcp*‐Co *100*/*002* region; however, the low‐intensity *200 fcc*‐Co^0^ peak offers unambiguous proof of the appearance of *fcc*‐Co^0^ (Figure 7a). This finite *fcc‐*Co° fraction continues to form up to the highest temperature investigated (425°C). To further explore this effect, another fresh sample was investigated under the same conditions but heated to 600°C. Indeed, the fraction of *fcc*‐Co^0^ is more prominent at higher temperatures, the overall diffraction pattern remains dominated by *hcp*‐Co^0^, indicating that hcp‐Co^0^ reflections are still more prominent than those of *fcc‐*Co^0^ (Figure S9). It should be noted that a quantitative determination of phase fractions from the in situ GIXRD data for thin‐film samples is not reliable because the diffracted intensities are strongly affected by the limited and potentially non‐representative probed volume, as well as by preferred‐orientation effects in the thin films. Therefore, the discussion is intentionally restricted to qualitative trends in peak evolution and phase transformation to avoid over‐interpretation of the data.

**FIGURE 7:**
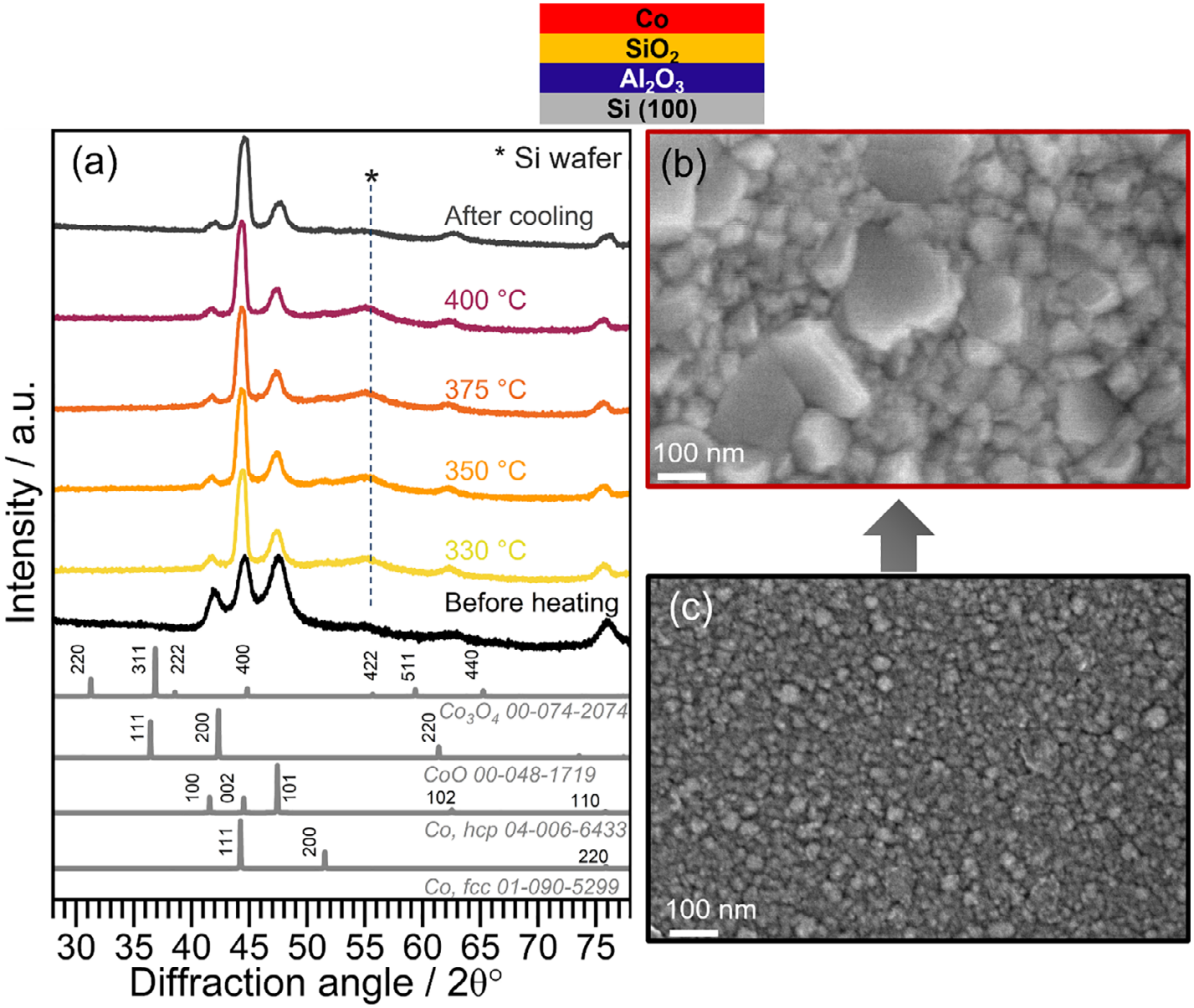
(a) Synchrotron‐based in situ GIXRD diffractograms of TFCat1 under reducing conditions (3% H_2_/97% He, 1 bar, ω = 1°, hν = 8 keV). (b) SEM micrograph of the TFCat1 sample after cooling down following the reduction treatment (∼14 h). (c) SEM micrograph of the as‐synthesized TFCat1 prior to the reduction treatment.

The transformation of the *hcp*‐to‐*fcc* structure in cobalt has been reported to be thermodynamic in nature, with *fcc* being the more stable structure above ca. 400°C, but kinetic constraints usually lead to the coexistence of both phases [46, 47, 48]. The crystalline phase of the cobalt, whether *hcp*‐ or *fcc*‐Co^0^, significantly influences the intrinsic activity in FTS, with *hcp*‐Co^0^ being more active than *fcc*‐Co^0^ [26, 27, 43]. Notably, *hcp*‐Co^0^ demonstrates greater intrinsic FTS catalytic activity and higher selectivity C_5+_, despite its propensity to oxidation, thus deactivation [49, 50]. On the contrary, the *fcc*‐Co^0^ generally exhibits relatively lower catalytic activity but influences the distribution of the product, however relatively more robust to oxidation [23, 24, 25, 43, 51, 52, 53]. Apparently controlled mixture of the two phases has been attributed to increased stability and altered selectivity, thus, implying that fine‐tuning of the ratio of the two phases may be the optimal strategy for directing catalyst performance. In the current example, the coexistence of the predominant *hcp*‐Co^0^ (with a minority *fcc*‐Co^0^) fraction might have the combination of preferred activity coupled with structural stability upon thermal cycling [25, 28, 43, 52]. Cooling from 400°C to RT (50°C/min) under a reducing environment, followed by exposure to air maintains the crystalline form obtained in the high‐temperature state with no sign of oxidation or reversion of the phase.

### 3.2 Phase Evolution of TFCat2

The as‐deposited TFCat2 shows the characteristic spinel Co_3_O_4_ diffraction pattern with the most prominent *311* peak and a minor contribution of *fcc*‐CoO (Figure 8a). Heating from RT to 330°C initiated reduction, as evidenced by a shift of the *311* reflection to lower angles and the appearance of the *hcp*‐Co^0^ *002* peak. With further heating to 350°C under reduction conditions for ca. 3.5 h, the spinel Co_3_O_4_ reflections disappeared, and the new diffraction pattern emerge that corresponds to *hcp*‐Co^0^. At 375°C, complete reduction was achieved, and further heating to 400°C sharpened the *hcp*‐Co^0^ reflections, indicating improved crystallinity. Upon cooling from 400°C to RT, a weak *fcc*‐Co^0^ *200* peak emerged at 51°, similar to TFCat1, suggesting that partial *hcp*‐to‐*fcc* phase transformation occurred at higher temperature under reduction conditions and is retained upon cooling (Figure 8a).

**FIGURE 8:**
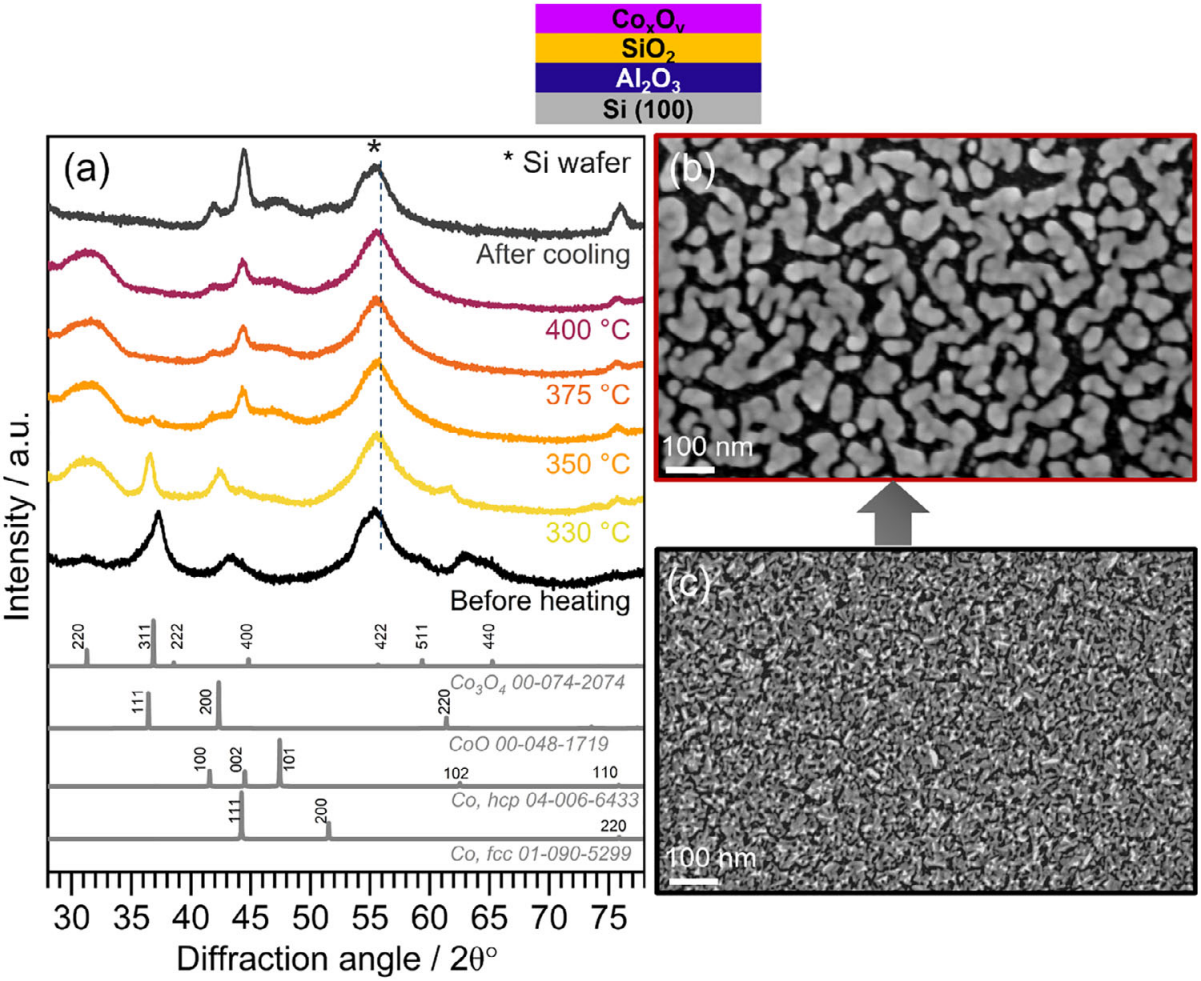
(a) Synchrotron in situ GIXRD diffractograms of TFCat2 under reducing conditions (3% H_2_/97% He, 1 bar, ω = 1°, hν = 8 keV). (b) SEM micrograph of the TFCat2 after cooling following the reduction treatment (∼14 h). (c) SEM micrograph of the as‐synthesized TFCat2 before the reduction treatment.

SEM micrographs confirmed that both TFCat1 and TFCat2 retained homogeneous film coverage after reduction at higher temperatures (Figures 7b,c and 8b,c). However, TFCat2 exhibited partial de‐wetting with apparent patches and discontinuities (Figure 8b). This occurs because of the removal of oxygen from the lattice of Co_x_O_y_ during reduction, causing large volumetric shrinkage, surface strain, and eventual film breakage [35]. De‐wetting is one of the ubiquitous degradation modes of thin‐film catalysts during thermal cycling and is particularly detrimental for patterned or structured supports, for which conformality is essential for retaining long‐term and stable catalytic activity. TFCat1, by virtue of starting in the metallic state, evades this oxygen‐removal process and is therefore beneficial in improving morphological stability even after ca. 14 h of reduction treatments. From a practical FTS standpoint, starting with the catalyst in the metallic phase (as in TFCat1) minimizes structural changes during activation, preserving the film integrity and conformality. While starting with an oxidic phase (Co_3_O_4_ and CoO, TFCat2) necessitates in situ reduction (to the active Co^0^ phase), which can introduce stresses that promote de‐wetting and loss of active area [23, 24, 30, 35, 52]. While oxide‐derived cobalt can, in some cases, yield smaller cobalt particles and higher dispersion post‐reduction, the mechanical stability trade‐off must be considered [35]. For structured catalytic supports (e.g., 3D nanostructures or hierarchical patterns), maintaining a continuous and conformal cobalt layer is crucial; thus, deposition directly into the desired metallic phase is preferable when stability is prioritized.

### 3.3 Fischer‐Tropsch Synthesis Catalytic Testing

The effect of the dynamic structural evolution of the synthesised thin‐film catalyst on Fischer‐Tropsch synthesis was probed under FTS operating conditions. Noteworthy, a blank experiment (with only SiC packing (without catalyst) was also conducted under FTS conditions identical to those employed for TFCat1 and TFCat2 (Figure S10). No measurable CO conversion was observed in the blank experiment, as confirmed by the absence of methane formation (Figure S11). This confirms that the observed CO conversion in the catalytic experiments can be attributed to the intrinsic catalytic activity of the catalyst under investigation. A lined‐out CO conversion level (*i.e*., CO conversion at steady state, cf. discussion below) of approximately 4.6% and 4.9% for TFCat1 and TFCat2 were obtained, respectively (Figure 9). As expected, the observed low CO conversion levels are due to the inherent low Co loadings in thin‐film catalysts [49]. Furthermore, the obtained lined‐out CO conversion levels can be considered statistically comparable given that both catalysts were evaluated under identical FTS operating conditions and with comparable cobalt film thicknesses (*cf*. STEM micrographs in Figures 5 and 6). At a weighted hourly space velocity (WHSV) of ca. 17.5 mol/g_cat_/h, the recorded lined‐out CO conversion levels corresponded to a CO consumption rate of *ca*. 0.81 mol/g_cat_/h and 0.86 mol/g_cat_/h over TFCat1 and TFCat2 samples, respectively.

**FIGURE 9:**
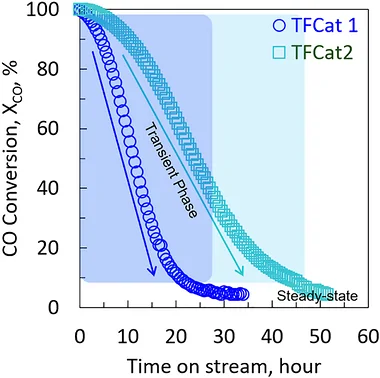
Time‐dependent CO conversion level obtained over the TFCat1 and TFCat2 samples, highlighting the distinct non‐steady‐state behaviour (as indicated by the arrows) of the two catalysts before reaching steady‐state.

Although both catalysts eventually reached similar steady‐state CO conversions, their transient behaviour differed markedly. At the beginning of the experiment, the reactor was pressurised with an inert gas (N_2_ in this case), which was gradually displaced by the syngas feed at a constant flow rate. Consequently, the apparent CO conversion initially started at nearly 100% and decreased progressively until N_2_ was fully replaced and reactor composition reached steady state with syngas. The blank experiment provides a baseline for assessing the intrinsic gas‐replacement dynamics of the reactor, decoupled from the effects of catalytic CO consumption (Figure S10). During this transient period (marked with arrows and highlighted in Figure 9), TFCat1 consistently exhibited lower CO conversion than TFCat2 at comparable reaction times and syngas composition, and reached steady state operation after ca. 20 h, whereas TFCat2 required ca. 40 h. The distinct transient behaviour of TFCat1 and TFCat2 may be associated with structural phase readjustments occurring during catalyst reduction, which require further investigations.

Comparison of the catalysts at iso‐conversion levels permits a more meaningful assessment of their intrinsic product selectivity distributions, independent of conversion‐related effects. While the CO conversion levels of the two catalyst systems are approximately comparable, pronounced differences were observed in their FT product selectivity distributions. The short‐chain hydrocarbon (HCs) products were classified and analysed according to their paraffinic and olefinic fractions. As the alcohol fraction represented an insignificant contribution to the overall product distribution, it was omitted from the subsequent discussion. The overall trend of the short‐chain hydrocarbons follows a typical Anderson−Schulz−Flory (ASF) plot, with C_2_ concentration being lower than C_3_ (Figure 10a). This is ascribed to the reabsorption of the highly reactive ethylene product/intermediate for secondary reactions, thus decreasing its concentration.

**FIGURE 10:**
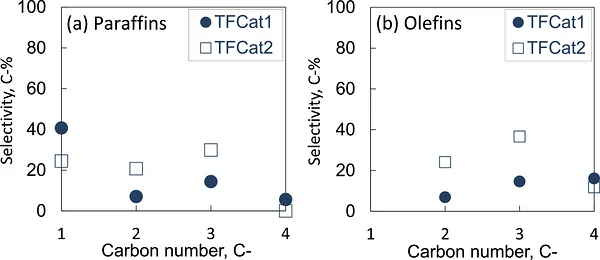
Selectivity profiles of (a) paraffins and (b) olefins over TFCat1 and TFCat2, highlighting the distinct product distributions of the two catalyst systems.

The methane selectivity obtained over TFCat1 and TFCat2 was approximately 41% and 25%, respectively. This disparity may suggest that TFCat1 exhibits a greater propensity for hydrogenation reactions than TFCat2. However, it should be noted that methane selectivity is often sensitive to CO conversion and can increase at both low and high conversion levels. Consequently, attributing the higher methane selectivity solely to intrinsic catalyst properties may not provide a complete representation of catalyst performance and should therefore be interpreted in conjunction with the broader product selectivity distribution. When considering the olefin fraction, overall, TFCat2 exhibited higher selectivity toward C_2_‐C_4_ olefins than TFCat1 (Figure 10b), as reflected by the olefin/paraffin (O/P) ratio of 1.040 and 1.68 for TFCat1 and TFCat2, respectively. This observation further supports the notion that hydrogenation reactions are more pronounced over TFCat1, resulting in (i) higher desorption rate, yielding higher methane selectivity and (ii) the preferential conversion of olefinic intermediates to their corresponding n‐paraffins.

Due to low yields, the liquid FTS products could not be reliably quantified; thus, qualitative data is presented in Figure 11. Consistent with the trends observed for the short‐chain hydrocarbons, TFCat1 produced a comparatively lighter paraffinic hydrocarbon distribution, with C_10_‐C_11_ representing the median carbon fraction and C_13_ being the highest detectable carbon number (Figure 11a). In contrast, TFCat2 yielded substantially heavier hydrocarbons, with C_17_‐C_20_ constituting the median fraction and carbon numbers extending up to C_27_ (Figure 11b). The pronounced shift toward heavier hydrocarbons over TFCat2 catalyst suggests enhanced chain‐growth probability (α) relative to TFCat1 catalyst. Conversely, the lighter product distribution obtained over TFCat1 catalyst may indicate a greater propensity for hydrogenation and chain‐termination reactions, thereby limiting hydrocarbon chain growth. This trend is primarily important for kerosene‐range HCs (C_8_‐C_16_) and TFCat1 shows higher tendency to provide product distribution more aligned toward kerosene production. Distinct product distribution by the two catalysts also highlights the importance of rational catalyst design where carefully tailored active species result in desired product distribution as shown in the case of TFCat1.

**FIGURE 11:**
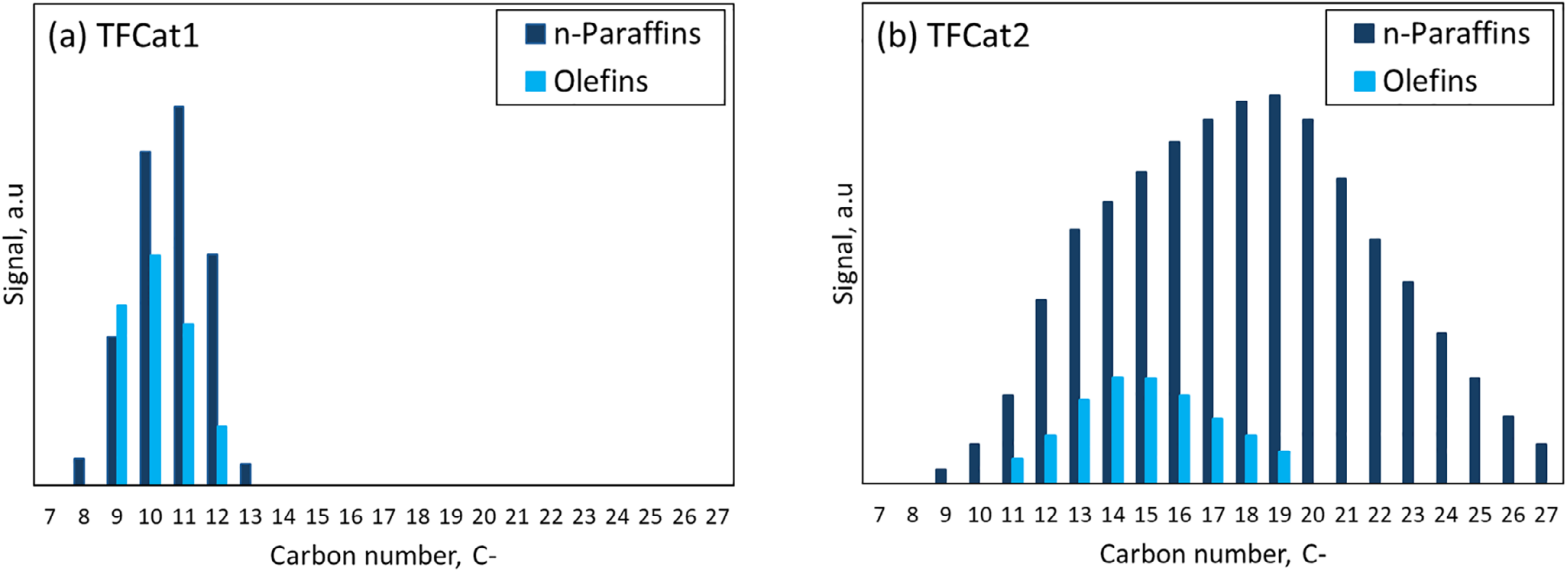
C_5+_ selectivity profiles for (a) TFCat1 and (b) TFCat2, highlighting the distinct product distributions of the two catalyst systems.

The surface structures of both spent catalysts were analyzed by SEM to observe the morphological changes induced by FTS testing. Both catalysts deposited on Al foil show a homogeneous film deposition and exhibit a certain degree of layering while retaining highly dense, continuous films after FTS testing, although with distinctly different morphologies. TFCat1, containing as‐deposited metallic cobalt, showed a typical grain‐like structure with no film dewetting with some defects mostly originating from the foil texture (Figure S12a). In contrast, TFCat2, with as‐deposited Co_x_O_y_, show a markedly different morphology. Although the film remained continuous without major dewetting, strongly widened grain boundaries developed within the surface, most likely due to restructuring during reduction and the removal of oxygen (Figure S12b). These distinct post‐reaction morphologies have a direct impact on the differences in catalytic activity and selectivity observed during FTS, as demonstrated by the FTS activity above. A more comprehensive understanding of the nature of the surface‐active sites would be highly beneficial for establishing clear structure–activity relationships, particularly in the context of thin‐film model catalysts.

## 4 Summary

Cobalt thin films with tailored chemical composition, crystalline phases and morphologies were fabricated in metallic (hexagonal and face centred cubic Co^0^), rock salt CoO and spinel‐Co_3_O_4_ phases via physical vapor deposition. The phase composition and surface architecture of these films were systematically tuned by changing the sputtering parameters and post‐deposition annealings. The as‐deposited films were homogeneous and stable under both ambient and vacuum conditions, with their crystallinity significantly enhanced upon thermal treatment in vacuum or reducing environments. Under reduction conditions, cobalt films can be engineered to predominantly *hcp*‐Co^0^, with minor *fcc*‐Co° contributions. Oxidation in air of the 40 nm thick Co° film at 350°C yields phase‐pure *spinel* Co_3_O_4_ with a substantial morphological transformation into rod‐ and needle‐like structures, which are distinctly different from those obtained via the direct reactive sputtering of cobalt oxide (*i.e*., pyramid‐ or needle‐shaped structures). This two‐step synthesis route, that is, the deposition of metallic cobalt by nonreactive magnetron sputtering (MS) followed by oxidation in air, demonstrates superior reproducibility of pure spinel phase than direct oxide deposition via reactive MS. In situ GIXRD further confirmed that the oxidation of the Co^0^ thin film initiated at ∼330°C and reached full Co_3_O_4_ conversion within approx. 3.5–4 h at 350°C. A thin‐film catalyst system mimicking commercial Fischer–Tropsch powder catalysts was developed in two architectures: Si/Al_2_O_3_/SiO_2_/Co^0^ (TFCat1) and Si/Al_2_O_3_/SiO_2_/Co_x_O_y_ (TFCat2). In situ synchrotron‐based GIXRD studies under reduction conditions revealed temperature‐dependent phase transformations in these stacks, underscoring the superior stability of metallic Co compared to spinel‐Co_3_O_4_. Although TFCat1 and TFCat2 exhibited statistically comparable lined‐out CO conversion levels, TFCat1 showed higher selectivity toward kerosene range hydrocarbons compared to TFCat2 suggesting differences in the availability of active sites and/or the rate of structural evolution under reaction conditions between TFCat1 and TFCat2. Starting with metallic Co films lead to a markedly reduced dewetting under reduction conditions. These findings demonstrating that the direct deposition of metallic cobalt maybe the optimal strategy when the catalytically active phase is metallic, as it minimizes dewetting and preserves the active surface coverage resulting in selective transformation to kerosene. The demonstrated ability to modulate the phase, morphology, and stability of such thin‐film systems offers a promising foundation for their deployment in advanced and durable catalytic technologies.

## Conflicts of Interest

The authors declare no conflicts of interest.

## Supporting information




**Supporting File**: smll75333‐sup‐0001‐SuppMat.docx.

## Data Availability

The data that support the findings of this study are available from the corresponding author upon reasonable request.
