# Supplementary material for: Engineering Cobalt‐Based Thin Films for Fischer–Tropsch Synthesis
Source: Small. 2026 Aug 24;22(60):e75333. doi: 10.1002/smll.75333 (PMC13711477; doi:10.1002/smll.75333)
Supplement: Supplementary file 1 [file SMLL-22-e75333-s001.docx]

***Supporting Information***

**Engineering Cobalt-based Thin Films for Fischer–Tropsch Synthesis**

*Muhammad Hamid Raza^1*^, Avela Kunene^1^, Alexander Steigert^1^, Athanasios Skaltsogiannis^2^, Catalina E. Jiménez^2,3^, Florian Ruske^4^, Ali Shan Malik^1^, René Schwiddessen^5^,* *Judith Helena Mettke^6^*, *Johannes Frisch^2,3^, Regan G. Wilks^2,3^, Erik Reichelt^6^, Marcus Bär^2,3,7,8^, Rutger Schlatmann^1^ and Daniel Amkreutz^1^*

1. PVcomB, Helmholtz-Zentrum Berlin für Materialien und Energie GmbH (HZB), Berlin, Germany
2. Department of Interface Design, HZB, Berlin, Germany
3. Energy Materials In-Situ Laboratory Berlin (EMIL), HZB, Berlin, Germany
4. Department Active Materials and Interfaces for Stable Perovskite Solar Cells, Helmholtz‐Zentrum Berlin für Materialien und Energie (HZB), Berlin, Germany
5. Department Structure and Dynamics of Energy Materials, Helmholtz-Zentrum Berlin für Materialien und Energie (HZB), Berlin, Germany
6. Fraunhofer Institute for Ceramic Technologies and Systems IKTS, Dresden, Germany
7. Helmholtz-Institute Erlangen-Nürnberg for Renewable Energy (HI ERN), Department of X-ray Spectroscopy at Interfaces of Thin Films, Berlin, Germany
8. Friedrich-Alexander-Universität Erlangen-Nürnberg (FAU), Department of Chemistry and Pharmacy, Erlangen, Germany

*[hamid.raza@helmholtz-berlin.de](mailto:hamid.raza@helmholtz-berlin.de)

**Table S1**. Deposition conditions and details of the cobalt-based thin films prepared by DC reactive and non-reactive magnetron sputtering (MS).

**Figure S1**. Visualization of the experimental design for the heat treatments and scans executed during the *in-situ* GIXRD measurements under reducing conditions (3% H₂/97% He, 1 bar, ω = 1°, hν = 8 keV) recorded at the KMC-2 beamline using a 2D Bruker Vantec 2000 detector and an X-ray transparent furnace (Anton Paar DHS 1100). All heating, cooling process and the measurements in the shaded area were done in H_2_.

**Figure S2**. *In-situ* GIXRD diffractograms for the sample S1, as deposited and after annealing in UHV at 600 °C.

**XPS: Atomic ratio calculation**

Oxygen to cobalt atomic ratios were calculated using Equation 1, where $I$ is the intensity (area) of the core level peak, $\sigma$ is the photoionization cross section, $\lambda$ is the inelastic mean free path (IMFP) of the ejected electrons in the sample after photoionization and $T$ is the transmission function of the electron analyzer which is a function of the detected electrons kinetic energy for the same instrument parameters (i.e pass energy and slit size). The area of each core level peak was calculated using CasaXPS software[1] from the raw experimental data after subtracting a Shirley-type background (without peak fitting). Cross section values were taken from tables provided by Scofield[2] for Al K_α_ excitation energy and ignoring angular distribution effects as the geometry of the apparatus was such that the angle between the X-ray source, the sample and the analyser was approximately equal to the magic angle (54.7°). The inelastic mean free path ($\lambda$) values were calculated through the empirical TPP-2M equation[3, 4] via the SESSA software.[5]

$Atomic ratio \left( O/{Co} \right)=\frac{I_{O,m}}{I_{Co,n}}\cdot\frac{\sigma_{Co,n}\cdot\lambda_{Co,n}\cdot T_{O,m}}{\sigma_{O,m}\cdot\lambda_{O,m}\cdot T_{Co,n}}$	(1)

The subscripts $m$ and $n$ stand for the O and Co core level peaks respectively, selected for the area calculation. Atomic ratios O/Co for R5 and R3 were calculated selecting the Co 3s and O 2s core level spectra (**Figure S3**). Their similar photoelectron kinetic energies allow for neglecting the IMFP and transmission function correction terms in equation 1. Co 2p and O 1s core levels were also used for comparison (**Figures 4** and **S4**). For the sample NR1, the lines O 1s and Co 2p were preferred due to the very small amount of O, and thus the low O 2s peak intensity. When the Co 2p and O 1s lines were used for atomic ratio calculations, the intensity values were corrected using the following transmission function:

$$T \left( KE \right)=0.03+0.001764\times\left( KE \right)-2.463\cdot{10}^{-6}\times\left( KE \right)^{2}+2.608\cdot{10}^{-9}\times\left( KE \right)^{3}$$

$-1.361\cdot{10}^{-12}\times{(KE)}^{4}+2.567\cdot{10}^{-16}\times{(KE)}^{5}$	(2)

where $(KE)$ is the kinetic energy in eV of the photoemitted electrons from the corresponding core level. The transmission function was generated by comparing a survey spectrum of clean Ag measured in our system to an intensity-corrected reference spectrum. All the values of the parameters in Eq. 1 used for the atomic ratio calculation are summarized in **Table S2**. It was observed that using the Co 2p/O 1s pair for the O/Co atomic ratio calculation resulted in lower values by around 20% compared to those derived when Co 3s and O 2s lines were selected. This underestimation probably stems from the wide O 1s loss peak at high binding energies that was not measured and thus not included in the peak area calculation.

**Figure S3.** Al K_a_ excited Co 3s (A) and O 2s (B) detail spectra for the samples R5 and R3 along with a Shirley-type background used to calculate the intensity (area) value that was subsequently introduced to equation 1 for the atomic ratio calculation.

**Table S2**. Parameter values used in Eq. 1 for the atomic ratio calculation.

|  | **Line** | **IMFP** (nm) * | $\boldsymbol{\sigma}$ (-) ** | $\boldsymbol{T}$(-)*** | $\boldsymbol{I}$ (cps) | **O/ Co atomic ratio** (-) |
| --- | --- | --- | --- | --- | --- | --- |
| **NR1** | Co 2p | 1.20 | 19.16 | 0.669 | 56032 | 0.04 |
|  | O 1s | 1.48 | 2.93 | 0.806 | 332 |  |
| **R5** | Co 2p | 1.42 | 19.16 | 0.669 | 43790 | 0.78 |
|  | O 1s | 1.75 | 2.93 | 0.806 | 5315 |  |
|  | Co 3s | - | 0.82 | - | 1554 | 1.01 |
|  | O 2s | - | 0.14 | - | 270 |  |
| **R3** | Co 2p | 1.42 | 19.16 | 0.669 | 17097 | 0.80 |
|  | O 1s | 1.75 | 2.93 | 0.806 | 2150 |  |
|  | Co 3s | - | 0.82 | - | 595 | 0.96 |
|  | O 2s | - | 0.14 | - | 98 |  |

*Calculated for Co metallic medium for NR1 and for CoO medium for R5 and R3. For the Co 3s, O 2s lines was not considered due to similar photoelectrons kinetic energies. **Values relative to C 1s cross-section which equals to 1. ***For the Co 3s, O 2s lines was neglected due to similar photoelectrons kinetic energies.

**XPS peak fitting procedure**

CasaXPS software was used for peak fitting of the XPS measurements. In all cases a Shirley-type background was used. For fitting the Co 2p spectrum of sample NR1 (pure Co metal) a similar approach to that of Biesinger et al. was followed,[6] but for the whole core level energy range instead of only for the Co 2p_3/2_ line. Apart from the main photoemission peak two plasmon loss peaks were used for fitting each of the spin orbit-split components with an intensity ratio between the Co 2p_3/2_ and the corresponding Co2p_½_ peaks set equal to 2. The plasmon loss process has been previously justified experimentally via reflection electron energy loss spectroscopy (REELS) and suggested that it can be adequately described by two broad peaks positioned at around 3 and 5 eV above the main peak.[7] As Co 2p core level sits between two Co LMM Auger features (when Al Ka is used as excitation energy), a small Auger peak at binding energy of 776.5 eV was also included as suggested previously.[6] A Doniach-Sunjic (DS) line shape was selected for all the metallic Co 2p peaks due to their asymmetric character, which stems from excitations to the conduction band of the metal taking place as shake-up process during photoemission.[8] Apart from the intensity ratio between the spin-orbit split components that was fixed, all the other parameters such as the peak positions and the full width at half maximum (FWHM) value were fitted. The FWHM of the main photoemission peak for the Co 2p_½_ was 0.7 eV larger compared to that of the Co 2p_3/2_ component, mainly due to Coster-Kronig broadening effects, in agreement with the literature.[9] The R5 sample was assumed to be pure CoO after evaluating the survey spectrum and the O/Co atomic ratio. For fitting the Co 2p core level spectrum of CoO, three peaks were included for each spin-orbit split component.[10] Besides the main photoemission peak, a multiplet-splitting peak and a shake-up satellite peak are assigned. The former is due to coupling between the unpaired electron in the core after photoemission with the unpaired outer shell electron, which can result in more than one final states. The satellite peak has been attributed to O 2p*e_g_* to Co 3d*e_g_* electron excitations (shake-up process).[11] A Voigt function was used for all the CoO fitting peaks. All the peak fitting parameters are summarized in **Table S3**.

**Table S3.** XPS Co 2p peak fitting parameters

|  |  | NR1 (Co metal)* | R5 (CoO)** |
| --- | --- | --- | --- |
| Peak A | Area | A | A |
|  | Position (eV) | 778.11 | 779.77 |
|  | FWHM | 1.00 | 2.61 |
| Peak B | Area | A x 0.204 | A x 0.366 |
|  | Position (eV) | 780.62 | 782.07 |
|  | FWHM | 3.80 | 2.43 |
| Peak C | Area | A x 0.302 | A x 1.07 |
|  | Position (eV) | 782.89 | 786.07 |
|  | FWHM | 3.80 | 6.69 |
| Peak D | Area | A x 0.5 | A x 0.5 |
|  | Position (eV) | 793.79 | 795.48 |
|  | FWHM | 1.70 | 3.10 |
| Peak E | Area | B x 0.5 | B x 0.5 |
|  | Position (eV) | 795.72 | 797.57 |
|  | FWHM | 7.54 | 2.68 |
| Peak F | Area | C x 0.5 | C x 0.5 |
|  | Position (eV) | 798.20 | 802.58 |
|  | FWHM | 7.54 | 5.194 |

* DS(0.3,200,10) asymmetric function was used for the metallic Co peaks. A Co LLM Auger peak with was also included at 776.45 eV. ** A Voigt function GL(30) was used for the CoO peaks

**Figure S4.** Al K_α_ excited XPS survey spectra of the three samples.

**Figure S5.** Al K_α_ excited Oxygen 1s spectra of the three samples (dots) along with their fits (solid lines).

**Figure S6.** (a) GIXRD diffractograms for the Co thin films (I) as deposited, (II) after oxidation in air at 350 °C. (b) SEM micrographs after the oxidation treatments for the cobalt thin-film sample.

**Figure S7**. Synchrotron-based in-situ GIXRD diffractogram of TFCat1 under oxidation conditions (in air, ω = 1°, hν = 8 keV).

**Figure S8**. The corresponding EDX spectra of the elemental mappings shown in Figure 5 for TFCAT1 (a), and Figure 6 for TFCAT2 (b). The copper signal originates from the copper TEM grid, as the EDX spectra and HAADF-STEM EDX were acquired from a lamella mounted on a copper grid.

**Figure S9**. Synchrotron-based in-situ GIXRD diffractograms of TFCat1 under reducing conditions (3% H₂/97% He, 1 bar, ω = 1°, hν = 8 keV).

**Figure S10**. Time-dependent CO conversion level obtained over the black reactor, TFCat1 and TFCat2 samples, highlighting the distinct non-steady-state behaviour of the two catalysts before reaching steady-state operation.

**Figure S11**. Comparison of methane profiles obtained using TFCat1, TFCat2, and during blank tests under similar reaction conditions.

**Figure S12**. Scanning electron microscopy (SEM) micrographs of the spent samples: (a) TFCat1 and (b) TFCat2.

**References**

1.	Fairley, N., C.S. Ltd, and C.S.L. Staff, *CasaXPS Manual 2.3.15: Introduction to XPS and AES*. 2009: Casa Software, Limited.

2.	Scofield, J.H., *Hartree-Slater subshell photoionization cross-sections at 1254 and 1487 eV.* Journal of Electron Spectroscopy and Related Phenomena, 1976. **8**(2): p. 129-137.<https://doi.org/10.1016/0368-2048(76)80015-1>

3.	Powell, C.J., *Practical guide for inelastic mean free paths, effective attenuation lengths, mean escape depths, and information depths in x-ray photoelectron spectroscopy.* Journal of Vacuum Science & Technology A, 2020. **38**(2).10.1116/1.5141079

4.	Tanuma, S., C.J. Powell, and D.R. Penn, *Calculation of electron inelastic mean free paths (IMFPs) VII. Reliability of the TPP-2M IMFP predictive equation.* Surface and Interface Analysis, 2003. **35**(3): p. 268-275.<https://doi.org/10.1002/sia.1526>

5.	Wolfgang S.M. Werner, W.S., Cedric J. Powell b, *NIST National Standard Reference Data Series Simulation of Electron Spectra for Surface Analysis ( SESSA )*. 2024.

6.	Biesinger, M.C., B.P. Payne, A.P. Grosvenor, et al., *Resolving surface chemical states in XPS analysis of first row transition metals, oxides and hydroxides: Cr, Mn, Fe, Co and Ni.* Applied Surface Science, 2011. **257**(7): p. 2717-2730.<https://doi.org/10.1016/j.apsusc.2010.10.051>

7.	Grosvenor, A.P., S.D. Wik, R.G. Cavell, and A. Mar, *Examination of the Bonding in Binary Transition-Metal Monophosphides MP (M = Cr, Mn, Fe, Co) by X-Ray Photoelectron Spectroscopy.* Inorganic Chemistry, 2005. **44**(24): p. 8988-8998.10.1021/ic051004d

8.	John F. Moulder, W.F.S., Peter E. Sobol, and Kenneth D. Bomben, *Handbook of X Ray Photoelectron Spectroscopy*. Vol. 255. 1995.

9.	Nyholm, R., N. Martensson, A. Lebugle, and U. Axelsson, *Auger and Coster-Kronig broadening effects in the 2p and 3p photoelectron spectra from the metals 22Ti-30Zn.* Journal of Physics F: Metal Physics, 1981. **11**(8): p. 1727.10.1088/0305-4608/11/8/025

10.	Lykhach, Y., S. Piccinin, T. Skála, et al., *Quantitative Analysis of the Oxidation State of Cobalt Oxides by Resonant Photoemission Spectroscopy.* The Journal of Physical Chemistry Letters, 2019. **10**(20): p. 6129-6136.10.1021/acs.jpclett.9b02398

11.	Kim, K.S., *X-ray-photoelectron spectroscopic studies of the electronic structure of CoO.* Physical Review B, 1975. **11**(6): p. 2177-2185.10.1103/PhysRevB.11.2177
